# Skeletal Anchorage as a Therapeutic Alternative for Mandibular Second Molar Impaction: A Prospective Case–Control Study

**DOI:** 10.3390/dj12110359

**Published:** 2024-11-11

**Authors:** Martina Mezio, Federica Altieri, Michele Cassetta

**Affiliations:** Department of Oral and Maxillofacial Sciences, “Sapienza” University of Rome, 00161 Rome, Italy; federica.altieri@aslroma1.it (F.A.); michele.cassetta@uniroma1.it (M.C.)

**Keywords:** impacted mandibular molars, impacted tooth, miniscrew, brass wire, TADs

## Abstract

**Background:** The treatment of mandibular second molar (MM2) impaction presents a challenge for orthodontists and requires a surgical–orthodontic approach. This study aims to compare the effectiveness of two techniques for treating impacted MM2: a traditional technique using brass wire and a technique employing skeletal anchorage. **Methods:** Twelve MM2 with mesio-angular impaction, with an inclination angle between 25° and 40° and an impaction depth between 4 and 10 mm, were selected and randomly divided into two treatment groups. Patients in Group A were treated using the traditional brass wire technique, while those in Group B underwent treatment with a skeletal anchoring technique that utilized a miniscrew positioned in the retromolar region and an elastic sling chain. For both groups, treatment time and the influence of the disimpaction technique on oral health-related quality of life (OHRQoL) were evaluated using the short-form Oral Health Impact Profile (OHIP-14). **Results:** The results indicated an average treatment time of 168.67 ± 52.32 days for Group A and 76 ± 10.17 days for Group B, with a statistically significant difference (*p*-value = 0.0002). Regarding the impact on the patients’ OHRQoL, Student’s *t*-test did not reveal a statistically significant difference between the two groups at 3 and 7 days of follow-up. **Conclusions:** Both techniques are considered effective for the treatment of impacted MM2 (angulation 25–40°, depth 4–10 mm). The use of skeletal anchorage significantly reduces treatment times without negatively affecting OHRQoL. The results of this study should be confirmed by further studies with larger sample sizes.

## 1. Introduction

The absence of a tooth in the oral cavity beyond the physiological time for its eruption, with the simultaneous loss of its eruptive potential, is known as “impaction”. It most commonly affects third molars, followed by maxillary canines and mandibular second premolars (MM2). It occurs in MM2 with a prevalence of 0.05% to 2.3% [1,2,3,4]. MM2 is important for the normal development of dentition and facial growth. Together with the first molar, it plays a substantial role in completing occlusion and determining a correct vertical dimension. Its impaction negatively influences both functional and aesthetic aspects [5]. The etiology of MM2 impaction is highly controversial and includes systemic, local, and genetic factors, with unilateral impaction being more common than bilateral impaction. The main systemic and local factors causing MM2 impaction are craniofacial morphology, anterior or posterior crowding, dentoalveolar discrepancy, periodontal membrane disorder, and failure of the eruption mechanism. The eruption of the MM2 requires the guidance of the mandibular first molar (MM1) roots, and excessive space between MM1 and MM2 may allow for the mesial angulation of the developing MM2 [6,7,8]. Furthermore, the use of orthodontic devices induces the distal inclination of the MM1 (lip bumper, lingual arch, or molar distalization devices) and increases the risk of impaction, especially when the initial angulation of the MM2 with respect to the MM1 is greater than 24° [9,10]. In the literature, MM2 impaction is classified into mesial, distal, and vertical based on its axial inclination, with mesial impaction being the most frequent [11,12]. Treatment, often interdisciplinary and surgical–orthodontic, is challenging for the orthodontist and early diagnosis is essential in reducing the risk of impaction and simplifying the treatment [13,14]. The poor prognosis of MM2 disimpaction is related to difficulties in anchoring. Over time, various treatment techniques have been proposed using fixed orthodontic appliances such as springs and loops of different shapes and sizes. Modern techniques such as employing miniscrews and skeletal anchoring have facilitated the treatment of impacted second molars and reduced undesired dentoalveolar effects [15,16].

The present clinical study was designed to compare two techniques for the treatment of impacted mandibular second molars: a traditional technique with brass wire and an innovative technique of skeletal anchorage.

The primary objective was to evaluate the differences in treatment time between the two techniques.

The secondary objective was to evaluate the impact of the two techniques on oral health-related quality of life (OHRQoL).

The hypotheses of the study were that there are no significant differences in treatment time between the two methods and that the skeletal anchoring technique does not significantly impact the patient’s OHRQoL when compared to the brass wire technique (null hypothesis).

## 2. Methods

This prospective clinical study with parallel cohorts was conducted at the Department of Oral and Maxillo-facial Sciences, Sapienza University of Rome, from January 2020 to December 2023.

The MM2 was considered impacted when it did not erupt within the physiological period or when it was evident that spontaneous eruption was impossible.

The study followed the CONSORT and the STROBE guidelines (Strengthening the Reporting of Observational Studies in Epidemiology), and the ethical principles of the Declaration of Helsinki were respected. Informed consent was obtained from the guardians of all the study participants.

The diagnosis was based on clinical and radiographic assessments (orthopantomography). The Ethics Committee of the “Umberto I” Polyclinic of Rome was informed of the study and granted its approval (#5951).

The subjects who met the following inclusion criteria were selected:

- Caucasian individuals aged > 12 years.
- Pre-treatment orthopantomographic examination with 1:1 magnification ratio.
- Good oral hygiene.
- Absence of previous orthodontic treatment.
- Absence of systemic diseases and syndromic conditions.
- Unilateral or bilateral impaction of the mandibular second molar.
- MM2 with mesio-angular impaction, having an angle of inclination between 25° and 40° and depth of impaction between 4 and 10 mm.

The angulation of the lower permanent second molar was measured on the panoramic radiograph as described by Evans [4]. Two lines were drawn connecting the tips of the cusps of the first and second molars; from these, two perpendicular lines representing the long axes of the teeth were traced. The angle formed between the long axes of the first and second molars was measured, representing the impaction angle of the second molar.

**Figure 1:**
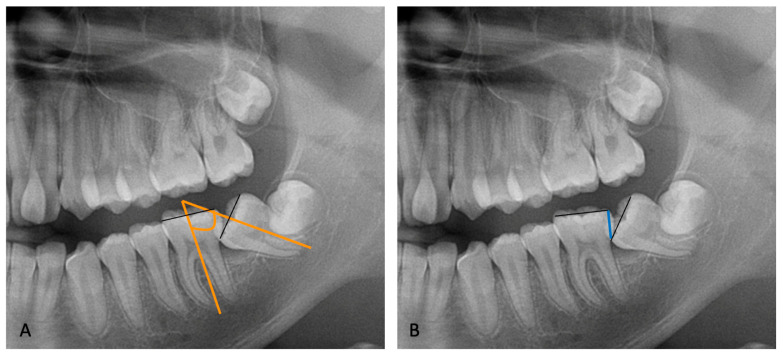
Orthopanoramic evaluation of angle (**A**) and depth (**B**) of impacted MM2.

The impacted depth was measured, as described by Fu et al. [17], as the vertical distance from the distal marginal ridge of the first molar to the mesial marginal ridge of the impacted MM2 (Figure 1).

To calculate the sample size, the following formula was used:

$$n=\frac{2{\left(Z_{\alpha }+Z_{1-\beta }\right)}^{2}\sigma^{2}}{\Delta^{2}}$$

Setting a significance level at alpha = 0.05 and a power level at 1 − *β* = 0.80, *σ* is the standard deviation of the treatment time, which is 52.53; and Δ represents the difference between the average treatment time of the control (Group A) (168.67) and study group (Group B) (76). Applying the formula, it was established that 4 (precisely 3.94) subjects per group were sufficient to obtain reliable results.

Twelve mesio-angularly impacted MM2 were selected and randomly divided into two treatment groups: the first group received the traditional disimpaction treatment with brass wire (Group A), while the second group was treated with a skeletal anchorage (Group B). A coin toss was used for the allocation to the two groups. To minimize potential errors by reducing a source of bias, all the subjects were treated by a single researcher (MC) experienced in both orthodontics and oral surgery.

The primary outcome was to determine the treatment time using the two types of disimpaction techniques. The secondary outcome was to determine the influence of the disimpaction technique used on oral health-related quality of life (OHRQoL) using the short-form Oral Health Impact Profile (OHIP-14).

For the surgical procedure, all the subjects in both groups were prescribed antibiotic therapy of 1 g of amoxicillin (Amoxicillin ABC 1 g; ABC Farmaceutici, Ivrea, Turin, Italy) one hour before the procedure and then twice a day for the next 5 days. Additionally, an ibuprofen tablet was administered one hour before the procedure for pain control (Brufen 600 mg; Abbott, Aprilia, Latina, Italy). The surgical phases involved the use of local infiltrative anesthesia using mepivacaine along with epinephrine in a ratio of 1:100,000 (Optocain 20 mg/mL, Molteni, Italy).

**Figure 2:**
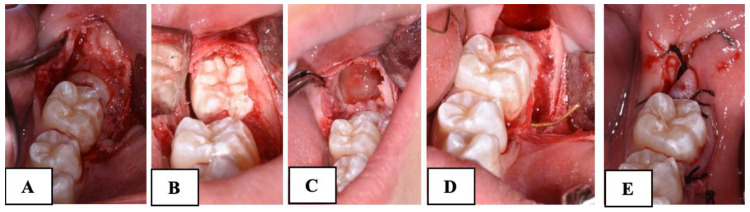
Surgical procedure with brass wire used in Group A subjects. (**A**): mucoperiosteal flap and the exposure of the impacted second molar; (**B**): the exposure of the third molar; (**C**): the germectomy of the third molar; (**D**): the application of brass wire; (**E**): suture.

A mucoperiosteal flap was elevated without releasement incisions, which extended from the mandibular first molar to the retromolar area on both the buccal and lingual sides. In all the patients of both groups, the procedure involved the exposure of the crown of MM2 and germectomy of the third molar. In the subjects treated with the traditional technique (Group A), a gentle luxation of MM2 was performed and a 0.6 mm double-twisted brass wire was inserted under the contact point between the first and second mandibular molars (Figure 2).

**Figure 3:**
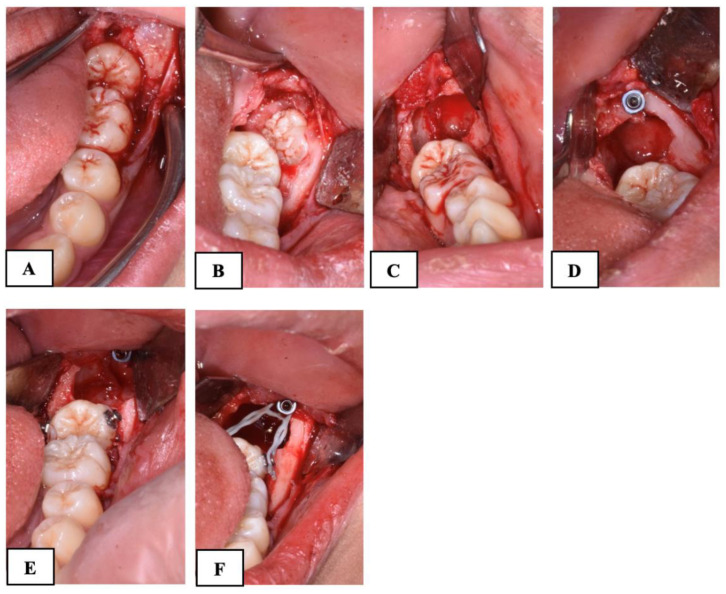
Surgical procedure using skeletal anchorage in Group B subjects. (**A**): mucoperiosteal flap and the exposure of the impacted second molar; (**B**): the exposure of the third molar; (**C**): the germectomy of the third molar; (**D**): miniscrew insertion; (**E**): the application of orthodontic brackets on the buccal and lingual surface of the mandibular second molar; (**F**): the application of the elastic chain.

In the subjects treated with skeletal anchorage (Group B), after the germectomy of the third molar, a self-drilling miniscrew (BENEfit, psm, Tuttlingen, Germany) was inserted in the retromolar area just distal to the post-extraction socket of the third molar using a surgical contra-angle (iSD900; NSK, Tochigi, Japan) with torque control (maximum torque set at 40 N/cm). The insertion site and angle, as well as the length and diameter of the miniscrew, were planned before the procedure through measurements from orthopantomography. After the miniscrew insertion, two orthodontic brackets were applied to the molar crown, one on the vestibular surface and one on the lingual surface. Finally, an elastic chain was attached to the miniscrew head and the orthodontic brackets to create an elastic sling. Once the primary miniscrew stability and the effectiveness of the elastic sling traction (the elastic chain was elongated to about twice its original length) were both verified, the surgical site was completely sealed with submerged traction using sutures (Figure 3).

**Figure 4:**
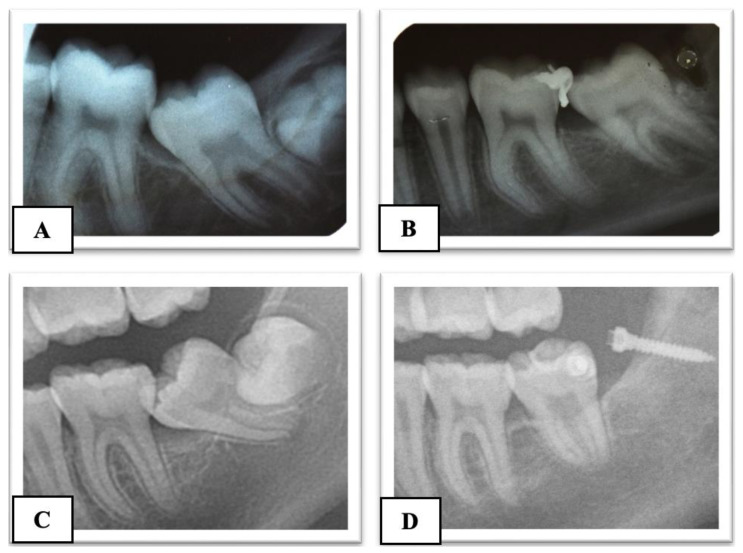
Pre-treatment (**A**) and post-treatment (**B**) X-ray of Group A subjects treated with brass wire; pre-treatment (**C**) and post-treatment (**D**) X-ray of Group B subjects treated with skeletal anchorage.

For the subjects treated with the traditional brass wire technique (Group A), follow-up visits were scheduled every 20 days, during which wire activations were performed until the uprighting of the MM2 was complete. In contrast, the Group B subjects had monthly follow-up visits until the complete uprighting of the MM2. The difference in the follow-up between the two groups is due to the need to periodically activate the brass wire to ensure the continuous movement of the MM2. In Group B, a longer follow-up was implemented because it was not necessary to reactivate the elastic chain. However, it remained important to monitor the patient for any potential complications, such as mini-implant failure or the detachment of the orthodontic button. In all cases treated with skeletal anchorage, there was no need to replace or reactivate the elastic chain, significantly reducing discomfort for the subjects. No miniscrew stability issues were observed in the Group B participants. The treatment was considered completed when the mesial marginal ridge of MM2 had passed the point of contact with the distal marginal ridge of MM1. The treatment time was recorded. A radiographic control was made before removing the orthodontic appliance in both groups (Figure 4). The advantages and disadvantages of the two techniques are shown in Table 1.

**Figure 5:**
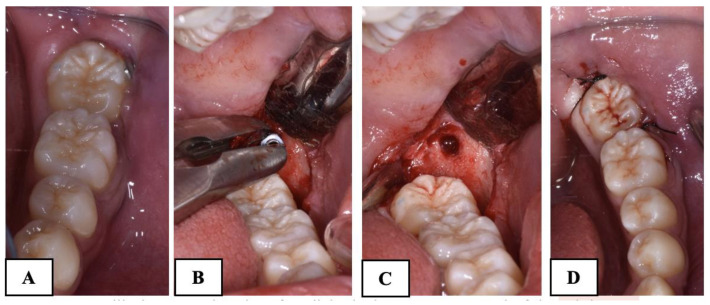
(**A**): Mandibular second molar after disimpaction; (**B**,**C**): removal of the miniscrew; (**D**): suture.

The brass wire, miniscrew, and orthodontic brackets were removed at the end of the treatment (Figure 5).

**Figure 6:**
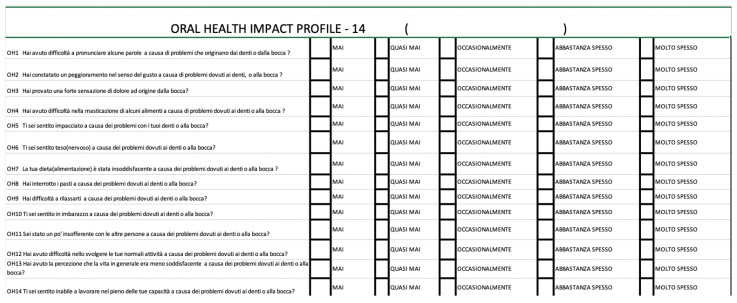
Italian version of OHIP-14.

The oral health-related quality of life (OHRQoL) tool is used to evaluate the impact of different oral health conditions on a person’s quality of life. This measurement allows for the comparison of the benefits and disadvantages of various treatments and provides a figurative representation of post-operative morbidity. Although several tools have been developed to measure OHRQoL, the most widely used and accepted is the Oral Health Impact Profile (OHIP), both in its complete form (OHIP-49) and the reduced form (OHIP-14). In this prospective study, the Italian version of OHIP-14 (Figure 6) was used, consisting of 14 questions representing seven dimensions of OHRQoL: functional limitation, physical pain, psychological discomfort, physical disability, psychological disability, social disability, and handicap.

The participants could respond to each question by choosing from five responses, each corresponding to a score ranging from 0 to 4:

0 = never;

1 = hardly ever;

2 = occasionally;

3 = fairly often;

4 = very often.

The level of OHRQoL is indicated by the overall score obtained with OHIP-14 (Figure 6), where higher scores indicate a greater negative influence of treatment on OHRQoL. The subjects completed the OHIP-14 questionnaire after receiving proper instructions before the disimpaction treatment (T0), and at three (T1) and seven (T2) days after the procedure.

All the data were recorded and stored in an Excel file and subjected to descriptive statistical analysis, reporting mean values and standard deviation. Student’s *t*-test was used to compare the treatment times in both groups and the influence of the two techniques on the OHIP-14 scores. For all the analyses, the *p*-value was set at 0.05. *p*-values ≤ 0.05 were considered statistically significant. The assumptions for conducting Student’s *t*-test were satisfied; specifically, the Shapiro–Wilk test for normality was performed, and all the *p*-values exceeded the threshold of 0.05. Therefore, we could not reject the null hypothesis of normality for all the analyzed variables. Additionally, Levene’s test for homogeneity of variance was conducted, and all the *p*-values were above the threshold of 0.05. Thus, we could not reject the null hypothesis of equality of variances between the study and control groups for all the analyzed variables. Statistical analyses were conducted using RStudio and Microsoft Office Excel. The methodologies employed included descriptive and inferential statistics, utilizing hypothesis tests such as Student’s *t*-test, Shapiro–Wilk test, and Levene’s test.

## 3. Results

In this prospective parallel-cohort study, a total of 12 impacted mandibular second molars in 12 subjects, 9 females and 3 males, were treated. Among the 12 participants, 6 received treatment using the traditional technique with brass wire (Group A), and 6 were treated with the skeletal anchorage technique using miniscrews (Group B). The inclination angle and depth values of the study sample are described in Table 2.

The average treatment time was 76 +/− 10.17 days for Group B and 168.67 +/− 52.32 days for Group A. Student’s *t*-test showed a statistically significant reduced treatment time in the patients treated with skeletal anchorage (*p*-value = 0.0002) (Table 3).

Regarding the impact on the patient’s OHRQoL in the two groups, the OHIP-14 values worsened from T0 to T1 (day 3 post intervention) It is important to note that, for all the subjects analyzed, regardless of the assigned group, there was an improvement in the OHIP-14 values from T1 to T2. Student’s *t*-test did not show a statistically significant difference between the two groups at 3 and 7 days of follow-up (Table 4).

## 4. Discussion

The results of the present study show that the first null hypothesis was not confirmed. The treatment time in Group B (disimpaction with skeletal anchorage) was significantly shorter compared to Group A (disimpaction with brass wire). However, the secondary null hypothesis was confirmed: the use of skeletal anchorage did not have a significant impact on the oral health-related quality of life (OHRQoL) compared to the use of brass wire.

The management of impacted second molars is considered a difficult and unpredictable challenge for both the orthodontist and the oral surgeon.

There are many treatment options for an impacted mandibular second molar: extraction, orthodontic uprighting, surgical uprighting, and the surgical–orthodontic approach.

According to the literature, the use of the traditional technique with brass wire appears to be effective in the treatment of slightly inclined impacted second molars [18,19,20,21]. Moro et al. [18] found an excellent result in the uprighting of the impacted third molar using the brass wire technique, suggesting that this therapy can also be used to treat impacted molars that have mesial inclination.

Kupietzky [19] states that this technique is a simple procedure that can be used successfully in the cases of impacted molars with a moderate degree of inclination, without however precisely defining the meaning of such an expression. Terms such as “mild”, “moderate” and “severe” are often used in the literature; however, no author has defined exactly what angulation ranges they are associated with. Lau et al. [22] defined a “severe” impaction of the second molar based on radiographic examinations, while Sawicka et al. [23] described severe mesial inclination by observing orthopantomographies.

Classifying the degree of impaction enables the determination of the level of case complexity and facilitates the choice between extraction or the uprighting of the dental element. The impaction of MM2 can be classified based on the angulation into mesially or distally inclined or vertically positioned [13].

Pogrel [24] suggested that the degree of inclination of the impacted second molar should not exceed 90°, as the surgical uprighting of MM2 (with or without orthodontic forced eruption) with an angulation greater than 90° can compromise pulp vitality.

Perdigão et al. [20] used this technique with brass wire for the bilateral disimpaction of second permanent molars, solving the problem in four months for the right side and five for the left. The treatment time is similar to that found in this study, which found an average treatment time of 168.6 days or just over 5 months.

This study confirms that the traditional technique with the use of brass wire is a simple procedure that can be effectively used in the case of “moderately” impacted molars (degree of inclination between 25° and 40°; depth of impaction between 4 and 10 mm). Furthermore, this technique allows a direct approach without the use of complex orthodontic devices, without laboratory steps, at a low cost, and without anchorage requirements. However, there may be discomfort for the patient during the surgical phase of positioning the brass wire and possible inflammation of the interdental soft tissues. Regular follow-ups are also required due to the need to reactivate the brass wire ligature to allow continued action.

Today, thanks to the use of miniscrews, it is possible to reduce the treatment time for the impaction of MM2 by eliminating undesirable dentoalveolar effects. The skeletal anchorage technique used in the present study allows the exposure of the impacted tooth, placement of the miniscrew, extraction of the crown of the third molar, and activation of orthodontic traction in a single appointment.

Regarding the combined use of miniscrews and elastic chains, few studies similar to the current one are present in the literature. Miyahira et al. [12] treated an impacted mandibular second molar with a miniscrew and elastic chain (treatment time of 3 months). Although the technique seems to be predictable and fast, the difficulty in maintaining oral hygiene around the miniscrew could cause more discomfort to the patient. In contrast, in the present technique, the absence of an intraoral orthodontic device thanks to a submerged technique allows optimal control of oral hygiene and no functional limitation of chewing.

Enache et al. [25] described a case of the bilateral impaction of the lower second molar with a severe degree of inclination, successfully treated using a miniscrew as skeletal anchorage and orthodontic bracket. The treatment time was around 8 months, during which the orthodontic button detached from the surface of the second molar, causing discomfort to the patient due to the size of the orthodontic device in the retromolar region, especially in the initial period. In this study, the average treatment duration was just over two months; moreover, no detachment of the buttons occurred, significantly reducing the patient’s discomfort.

Lee et al. [26] described three cases of mesio-inclined impacted MM2: in the first and second case a miniscrew positioned between the first molar and the second premolar was used, and a tube was applied on the disto-buccal surface of the crown of the second molar. The treatment time was 5 months, but the spring had to be replaced every 4 weeks. In the third case, the impaction of a second molar was treated in 2 months using a miniscrew positioned in the retromolar area connected to a single orthodontic button by an elastic chain.

To increase retention, Mah et al. [27] proposed the use of two miniscrews. In relation to the present study, treatment with multiple mini-implants seems to require about twice the time, and the use of two mini-implants could cause more discomfort to the patient.

Giancotti et al. [28] used a miniscrew positioned in the retromolar region without creating a flap, and orthodontic traction was applied through a closed coil spring connected from the miniscrew to the orthodontic bracket placed on the crown of MM2. The treatment lasted 8 months, but a subsequent finishing phase with fixed orthodontic appliances was needed to align the roots. In this study, it was not necessary to perform a finishing phase to improve the positioning of the roots. The use of two brackets, one on the buccal surface and one on the lingual surface, likely facilitates better correction of the root position and improved control of rotations and extrusion.

Sbricoli et al. [29] treated 20 impacted mandibular molars using a miniscrew positioned distally to the extraction site of the third molar and a coiled spring stretched between the miniscrew and an orthodontic bracket placed on MM2. The average treatment duration was 10 months, during which mucosal hypertrophy around the orthodontic bracket was observed in 15% of cases due to the patient’s difficulty in maintaining adequate oral hygiene. Additionally, in 7 cases, further orthodontic treatment was needed to finalize the correct position of MM2.

In the present study, the average treatment time was 76 days (just over 2 months). During treatment, no reactivation of the elastic traction was necessary, resulting in a reduction in number of appointments and improved patient comfort. Additionally, no significant adverse events or side effects were recorded. In the present study, the miniscrew was inserted in the retromolar area, and two orthodontic brackets were placed on the crown of MM2, respectively, on the vestibular and lingual sides. An elastic chain was applied from the miniscrew to the two brackets to create an elastic sling. Compared to other studies, this study used two brackets and an elastic sling chain for the first time, which allowed for a more accurate and controlled direction and intensity of force. Compared to previously described techniques, this technique has the advantage of reducing the size of the orthodontic device (less invasive compared to the use of two miniscrews or miniplate), minimizing chair time, and improving patient comfort. The application of force through the elastic chain promotes the distalization of MM2, allowing for the control of the movement and side effects, thus facilitating the rapid disimpaction of the crown. The use of an elastic sling, anchored on the buccal and lingual side of the second molar, is useful for coronal derotation and reduces the risk of unwanted rotation during the disimpaction movement. In addition, the position of the miniscrew head and the elastic sling design allows the vertical control of MM2. The position of the miniscrew head at the level of the occlusal plane prevents the extrusion of the MM2 crown. In this way, it was possible to achieve a pure disto-inclination of MM2, without rotation and extrusion, eliminating the need for a multi-bracket orthodontic appliance at the end of treatment, compared to the brass wire technique, where there is less control of verticality and rotation.

Furthermore, the present procedure offers complete control of anchorage and prevents the unwanted movement of adjacent teeth [12,28]. In the technique described, the miniscrew was inserted freehand into the retromolar area, as this area usually has a sufficient quantity and quality of bone, eliminating the need for a CBCT scan. Nowadays, thanks to dedicated software that combines STL files of dental arches with DICOM images obtained from CBCT, the insertion of orthodontic miniscrew using a computer-guided surgery is possible.

The positioning of the miniscrews in the retromolar area, as also reported by Ki-Jun Kim et al. [30], is very effective in the treatment of impacted MM2 and offers the advantage of guaranteeing excellent primary stability thanks to its high bone density. Furthermore, the positioning of the miniscrews in this region presents important biomechanical advantages: it allows the application of force distal to the center of resistance of the MM2, facilitating vertical control and rotation during the uprighting phase.

Although it has been demonstrated that the germectomy of the mandibular third molar does not reduce treatment times for the disimpaction of the second molar [21], in this study its extraction was necessary as its presence would have forced the operator to position the miniscrew above the occlusal plane, resulting in an undesirable extrusion effect.

This study also examined the influence of the two techniques on the patient’s oral health-related quality of life (OHRQoL), using the Italian version of the OHIP-14. The post-operative course showed no statistically significant differences between the two groups. The worsening of the OHIP-14 scores from T0 to T1 and the improvement from T1 to T2 reflect the normal post-operative course of any surgical procedure, with a prevalence of symptoms in the first three days and clinical improvement in the following days. This aspect has not been previously evaluated by other studies in the literature, but the results of the present study show that the impact of the two techniques on the patient’s OHRQoL is similar with a clear deterioration on the third post-operative day. In both techniques, a significant improvement in the patient’s OHRQoL can be seen as early as day seven. These results demonstrate that patient discomfort is associated with the surgical procedure (third molar extraction and exposure of the impacted MM2) and not with the orthodontic technique used. In fact, the insertion of the miniscrew did not cause a deterioration in the patient’s OHRQoL. It should be noted that the new technique with skeletal anchorage does not require subsequent activations as with the traditional technique thus an overall lower impact of this technique on the patient’s oral health-related quality of life can be conjectured.

## 5. Conclusions

The results obtained in this study show that the skeletal anchoring technique, using a miniscrew positioned in the retromolar region and an elastic sling chain (using a submerged technique), represents a valid alternative for the treatment of impacted MM2 (angulation 25–40°, depth 4–10 mm). Compared to the traditional disimpaction technique with brass wire, the use of skeletal anchoring allows the treatment time to be significantly reduced. The average treatment time was 76 +/− 10.17 days for Group B (skeletal anchorage technique) and 168.67 +/− 52.32 days for Group A (brass wire).

The insertion of the miniscrew and orthodontic traction did not have a statistically significant negative impact on OHRQoL, which showed a similar trend in both groups. Patient discomfort was attributed to the surgical procedure rather than the orthodontic technique employed. Further studies with larger sample sizes, longer follow-up periods, or different patient demographics could help validate the current study’s conclusions.

Undoubtedly, an early diagnosis can prevent the impaction of these dental elements, allowing a more conservative therapeutic approach to be adopted. Consequently, radiographic follow-up is essential to evaluate the eruption process of the MM2 and promptly intercept any complications.

## Figures and Tables

**Table 1:** Advantages and disadvantages of two techniques.

Surgical–Orthodontic Approach with Brass Wire		Surgical–Orthodontic Approach with Skeletal Anchorage	
Advantages	Disadvantages	Advantages	Disadvantages
- Direct approach without the need for complex appliances - No laboratory phases - Low cost - No anchorage requirements	- Potential discomfort for the patient during the surgical phase of brass wire placement - Possible inflammation of the interdental soft tissues - Frequent follow-ups due to the need for regular reactivation of the brass wire to ensure continuous action	- Absence of an intra-oral orthodontic device (submerged technique) allowing optimal oral hygiene control - No functional limitations to chewing - Maximum anchorage with no dental side effects - No need for elastic traction reactivation during treatment, reducing the number of appointments and improving patient comfort - No laboratory phases	- Potential patient discomfort during miniscrew placement and removal (requires flap elevation) - Possible post-operative complications (orthodontic bracket detachment, miniscrew stability loss)

**Table 2:** Descriptive analysis of the patient cohort: mean, maximum (Max) and minimum (Min) values and standard deviation (SD), first quartile (25%), median (Med), and third quartile (75%).

			*n*	Average	Max	Min	SD	25%	Med	75%
Group A	Sex	Males	2							
		Females	4							
	Age			12.7	13.4	12.1	0.56	12.40	12.25	13.18
	Inclination angle			38.0	40.0	36.0	1.89	36.25	38.00	39.75
	Depth of impaction			6.5	6.5	6.5	0	6.50	6.50	6.50
Group B	Sex	Males	1							
		Females	5							
	Age			15.32	20.5	12.8	2.96	13.0	14.85	16.10
	Inclination angle			38.67	40.0	32.0	3.27	40.0	40.0	40.0
	Depth of impaction			8.35	9.5	6.5	1.30	7.38	8.80	9.40

**Table 3:** Treatment time.

	Group A	Group B	*p*-Value
Average treatment time in days	168.67	76	0.0002
Standard Deviation	10.17	27.68	

**Table 4:** Mean OHIP-14 values pre-treatment (T0) and at 3-day (T1) and 7-day (T2) follow-up and mean changes Δ T1-T0 and Δ T2-T1.

	OHIP-14 T0	OHIP-14 T1	OHIP-14 T2	Δ T1-T0	Δ T2-T1
Group A	2.8	21.2	8.8	+18.3	−12.3
Group B	8.2	19.50	12.0	+11.3	−7.5
*p*-Value	0.15	0.82	0.575	0.17	0.14

## Data Availability

The data presented in this study are available upon request from the corresponding author because an online database has not been created.
